# Bacterial cellulose biomaterials for the treatment of lower limb ulcers

**DOI:** 10.1590/0100-6991e-20233536-en

**Published:** 2023-05-11

**Authors:** GLÍCIA MARIA DE OLIVEIRA, ANTÔNIO OSCAR GOMES, JAIURTE GOMES MARTINS DA SILVA, ALBERTO GALDINO DA SILVA, MARIA DANIELLY LIMA DE OLIVEIRA, CÉSAR AUGUSTO SOUZA DE ANDRADE, ESDRAS MARQUES LINS

**Affiliations:** 1 - Universidade Federal de Pernambuco, Programa de Pós-graduação em Inovação Terapêutica - Laboratório de Biodispositivos Nanoestruturados, Departamento de Bioquímica - Recife - PE - Brasil; 2 - Universidade Federal de Pernambuco, Laboratório de Biodispositivos Nanoestruturados, Departamento de Bioquímica - Recife - PE - Brasil; 3 - Universidade Federal de Alagoas, Campus Arapiraca - Arapiraca - AL - Brasil; 4 - Hospital das Clínicas da Universidade Federal de Pernambuco, Departamento de Angiologia e Cirurgia Vascular - Recife - PE - Brasil

**Keywords:** Biocompatible Materials, Cellulose, Arterial Occlusive Diseases, Leg Ulcer, Materiais Biocompatíveis, Celulose, Doença Arterial Periférica, Úlcera da Perna

## Abstract

Chronic ulcers of the lower limbs are common and recurrent, especially in the elderly population, they are disabling injuries that generate a great socioeconomic burden. This scenario encourages the development of new, low-cost therapeutic alternatives. The present study aims to describe the use of bacterial cellulose in the treatment of lower limb ulcers. This is an integrative literature review, carried out in the PubMed and Science Direct databases by associating the descriptors, with the inclusion criteria being clinical studies in the last 5 years, available in full in English, Portuguese and Spanish. Five clinical trials were analyzed and the main therapeutic effects obtained in the experimental groups that used bacterial cellulose dressings were a reduction in the area of the wounds, one of the studies showed a reduction of 44.18cm^2^ in the area of the wound, the initial lesions measured on average 89.46cm^2^ and at the end of the follow-up, they had an average of 45.28cm^2^, since the reduction in pain and the decrease in the number of exchanges were advantages described in all groups that used the BS. It is concluded that BC dressings are an alternative for the treatment of lower limb ulcers, their use also reduces operational costs related to the treatment of ulcers.

## RESUMO

As úlceras crônicas de membros inferiores são comuns e recorrentes principalmente na população idosa, são lesões incapacitantes e que geram grande ônus socioeconômico. Tal cenário incentiva o desenvolvimento de novas alternativas terapêuticas e de baixo custo. O presente estudo tem como objetivo descrever o uso da celulose bacteriana no tratamento de úlceras de membros inferiores. Trata-se de uma revisão integrativa da literatura, realizada nas bases de dados PubMed e Science Direct por meio da associação dos descritores, tendo como critério de inclusão estudos clínicos nos últimos 5 anos, disponíveis na integra em inglês, português e espanhol. Cinco ensaios clínicos foram analisados e os principais efeitos terapêuticos obtidos nos grupos experimentais que utilizaram os curativos de celulose bacteriana foram redução da área das feridas, um dos estudos apontou uma redução de 44,18cm^2^ da área da ferida, as lesões iniciais mediam em média 89,46cm^2^ e ao término do acompanhamento apresentaram uma média de 45,28cm^2^, já a redução da dor e diminuição do número de trocas foram vantagens descritas em todos os grupos que utilizaram a CB. Conclui-se que os curativos de CB são uma alternativa para o tratamento das úlceras de MMII, sua utilização reduz também os custos operacionais relacionados ao tratamento de úlceras.

## INTRODUCTION

Chronic wounds or wounds that are difficult to heal are those that do not heal properly over a normally acceptable period of time^1^. The mean annual cost of a non-healing wound has been predicted to be US$ 23,300 per affected patient, ranging from US$ 1,800 to US$ 61,500^2^. The humanistic and economic burden of chronic wounds is underestimated and is increasing due to an aging population and the early onset of chronic diseases^3^.

Among chronic wounds, ulcers of the lower limbs stand out because they are an important public health problem, both in Brazil and in the world, with physiological, psychosocial, and cultural implications^4^. According to the Wound Healing Society, about 15% of the elderly in the USA are affected by chronic wounds, predominantly venous stasis ulcers, pressure ulcers, and diabetic foot ulcers^5^.

The most common etiology of these lesions is chronic venous insufficiency (CVI), applicable to approximately 80% of leg ulcer cases, occurring due to abnormal function of the venous system caused by valvular insufficiency, which may be related to obstruction of blood flow^6^ ^,^ ^7^. Up to two thirds of all leg ulcers will be of venous origin, with a prevalence of 1% to 3% in the general population^8^.

Ischemic wounds, on the other hand, arise because of the most severe manifestation of peripheral arterial disease (PAD) of the lower limbs, which frequently affects the foot and leg, and is associated with a high risk of limb loss^9^. The high cost associated with the treatment does not favor obtaining an effective cure for the disease, which can be explained by the lack of therapeutic standardization^10^.

Several polymeric materials are used for the treatment of chronic wounds and have advantages such as promoting growth factors, moisture retention, increased neovascularization, protection against microbial agents, and tissue adhesiveness. Their role as good wound healing agents essentially depends on their biodegradability, biocompatibility, non-immunogenicity, and mechanical properties^11^. Bacterial cellulose (BC) has recently drawn greater attention as the focus of numerous studies. BC is biosynthesized by various strains of bacteria from glucose monomers that generate a three-dimensional network of interconnected nanostructured cellulose fibers. It can be produced on a large scale by controlled fermentation^12^. BC is a non-toxic, biocompatible biomaterial made from biotechnological synthesis^11^ ^,^ ^13^. It has been studied in several areas of medicine and gained prominence for having adequate characteristics and promising results when used as a dressing and biological graft^14^ ^-^ ^16^. It has wide biomedical applicability, such as nail prosthesis, tympanic graft, in the postoperative period of hypospadias correction, in the treatment of pressure injuries, also presenting ideal properties as a dressing^15^ ^-^ ^18^. This integrative review (IR) aims to describe the healing effect of bacterial cellulose in the treatment of lower limb ulcers.

## METHODS

This study is about an Integrative Review (IR), which is a method that provides opportunities for the synthesis of knowledge through a systematic and rigorous process. The IR should be based on the same recommended principles of methodological rigor in research development^19^. For the synthesis of the study, we defined the objective, determined the inclusion and exclusion criteria, analyzed the studies found, and discussed the results. The research’s guiding question was, “Are bacterial cellulose-based biomaterials effective for the treatment of lower limb ulcers?”.

We searched the US Public National Library of Medicine (PubMed) and Science Direct databases by associating the terms “bacterial cellulose” and “ischemic wounds” or “cellulose biomembrane and ischemic wounds” or “bacterial cellulose and ulcers venous” or “cellulose biomembrane and ulcer venous”. Include work comprised clinical studies published between 2017 and 2022 that represent the applicability and recent advances in the use of cellulose-based biomaterials, available in full, in Portuguese, English, and Spanish. We excluded preclinical studies, reviews, abstracts, incomplete texts, and gray literature. Terms were combined in PubMed and Science Direct databases, resulting in the search strategy shown in the flowchart.

## RESULTS

We found 17 articles through the search strategies in the databases. After reading the titles, 14 studies remained, of which five were clinical trials and met the inclusion criteria. Table 1 brings the studies’ characteristics.

**Table 1:** Selected studies Characteristics.

Author and year	Type of study	Population characteristics	Follow-up time	Goals	Main results	Conclusions
Silva GL et al. (2021)	Randomized controlled clinical-intervention study	Wound type: Chronic venous ulcer. Population sample: 39 adults, regardless of sex. Groups: Experimental groups (EG) 19 patients, treated with BC dressing; Control group (CG) 20 patients.	Followed for 180 days, evaluated according to the MEASURE methodology.	Evaluate the effectiveness of treatment for chronic venous ulcer of the lower limbs.	In both groups, the wound area significantly decreased (p<0.001) and the healing rate was similar to the CG’s. The mean number of dressing changes in the EG was 18.33 ± 11.78, while in the CG it was 55.24 ± 25.81, p<0.001.	The dressing made of bacterial cellulose, gel, and associated film, by stimulating the epithelialization of the lesions, showed a significant reduction in the initial area, with a cure rate like that of the Rayon^®^ dressing, in addition to requiring less direct manipulation of the ulcers.
Maia AL et al. (2019)	Randomized clinical trial.	Wound type: Arterial ulcer/ischemic wound. Population sample: 24 patients after lower limb revascularization. Groups: Experimental Group (13 patients) treated with bacterial cellulose biopolymer film and gel. Control Group (11 patients) treated with essential fatty acids.	Followed for 90 days in weekly appointments to change dressings and evaluate healing process.	To evaluate the use of a bacterial cellulose biopolymer film and gel dressing in the treatment of patients with ischemic wounds undergoing revascularization of the lower limbs.	The reduction in the areas of ischemic wounds after 30 days was 4.3cm^2^ (55%) on average for the experimental group and 5.5cm2 (48.5%) for the control one (p>0.05). The complete healing rate at 90 days was 34.8%, 50% in the experimental group and 18.2% in the control one (p=0.053).	The bacterial cellulose biopolymer film associated with the gel can be used as a dressing in the treatment of ischemic wounds in patients undergoing revascularization of the lower limbs.
Author and year	Type of study	Population characteristics	Follow-up time	Goals	Main results	Conclusions
Cavalcanti LM et al. (2017)	Prospective, randomized, controlled study.	Wound type: chronic venous ulcer Population sample: 25 randomly assigned patients. Groups: Control group (11 patients) received dressings with triglyceride oil. Experimental group (14 patients) treated with BC membrane (14 patients).	Followed for a period of 120 days.	To evaluate the effectiveness of dressings with Bacterial Cellulose membrane in the treatment of venous ulcers of the lower limbs.	There was a reduction in wound area in both groups. There were no infections or reactions to the product in either group. Patients in the BC group showed a decrease in pain and earlier discontinuation of analgesics.	The BC membrane can be used as a dressing for the treatment of varicose ulcers of the lower limbs.
Colenci R et al. (2019)	Randomized, controlled clinical trial	Wound type: venous ulcer Population sample: Experimental group (25 patients with 37 ulcers in the biomembrane group). Control group (21 with 36 ulcers treated with collagenase)	Both groups received compression therapy. The primary outcome was ulcer area reduction at 90 days (T90).	To evaluate the efficacy and safety of cellulose biomembranes in comparison with the collagenase dressing for the treatment of venous ulcers.	In the primary outcome, there was a reduction in lesions’ area over time, but this difference was not significant between groups (at T90, p=0.66). Of the 73 ulcers, 19 ulcers healed, 12 (48%) in the biomembrane group and seven (33%) in the collagenase group (relative risk 1.4, 95% CI 0.7 3.0, p=0.30). There was no significant difference between groups.	The reduction of VU area as a function of time was similar in biomembrane and collagenase dressings, with no significant difference between groups. However, the biomembranes promoted early wound healing and increased vascularity compared with collagenase.
Zanoti MDU et al. (2017)	Quantitative, descriptive longitudinal study.	Wound type: venous ulcer, diabetic foot, and mixed wound. Population sample: 14 patients (10 women and 4 men).	Treatment was discontinued after complete healing or a maximum of 120 days, or when the patient changed his or her behavior at medical request or on his/her own accord.	To describe the development of bacterial cellulose coating with anti-inflammatory Ibuprofen (BC/Ibu) and to evaluate the healing process with its use in patients with chronic wounds.	There was a reduction in area and pain in nine lesions, total healing of three wounds, and debridement of devitalized tissue in five wounds with increased area. The use of the membrane decreased, pain, exudation, and facilitated performing the dressing.	BC/Ibu favored the healing process of patients with chronic vasculogenic wounds.

Of the analyzed studies, three (75%) used bacterial cellulose synthesized through the Zoogloea SP biodigestor and one (25%) used the bacteria Gluconacetobacter xylinus. In one (25%), ibuprofen was incorporated. The number of participants in each study ranged from 14 to 39. When analyzing clinical trials of lower limb UVC, three (75%) addressed venous ulcers (VU) and one (25 %), ulcers due to ischemic complications resulting from PAD.

As for the sociodemographic profile of the population, in the study by Silva et al. (2021) females were predominant in both groups (EG, 70%; CG, 73.7%), with a mean age of 62.41 ± 10.72 years. In the study by Cavalcanti et al. (2017), male participants constituted 54.5% of the control group and 50% of the experimental group, and the mean age of participants was 60 ±17 years in the control one, compared to 61 ± 14 years in the experimental group. The sample evaluated by Zanoti et al. (2017) consisted of 14 patients, 10 (71.50%) females and four (28.50%) males, age ranging between 43 and 86 years, with a mean of 64. As for the 24 patients evaluated by Maia et al. (2019), age ranged from 49 to 90 years (mean of 67.4) and 12 (50%) were female, with no variation regarding the sex.

Considering the proposed interventions, therapeutic effects prevailed in the experimental groups in all five studies, identified as reduction in wound area (Table 2), reduction in pain, and decrease in the number of exchanges

**Table 2:** Comparison of initial and final wound areas

Author and year	Experimental Group (EG) Area 1 of the lesion Initial (mean ± SD, cm^2^)	Experimental Group (EG) Area 1 of the lesion Final (mean ± SD, cm^2^)	Control Group (CG) Area 1 of the lesion Initial (mean ± SD, cm^2^)	Control Group (CG) Area 1 of the lesion Final (mean ± SD, cm^2^)
Silva GL et al. (2021)	62.86±89.46	31.06±45.28	37.99±48.30	17.67±31.19
Maia AL et al. (2019)	13.0±15.9	8.7±13.1	12.2±11.9	6.7±7.6
Cavalcanti LM et al. (2017)	54±57	54±49	50±59	36±27
Colenci R et al. (2019)	5.71 (1.52-14.90)**	6.23 (0-13.44)**	5.08 (1.52-10.08)**	(0.41-7.96)**
Zanoti MDU et al. (2017)	11.94*	11.34*	-	-

*No control group because it is a longitudinal descriptive study, the average being calculated based on the data described by the author. **Median area (cm ^*2*^ *).*

## DISCUSSION

### Lower limb ulcers

After analyzing the clinical trials of lower limb ulcers, four studies (80%) addressed venous ulcers and one (20%), ulcers due to ischemic complications resulting from PAD. This proportion is in line with the predominance of these lesions, since there are three different types of chronic lower limb ulcers, which can be venous, arterial, and mixed. Isolated venous insufficiency is the cause of 70-80% of chronic leg ulcers; venous and arterial ulcers, called mixed ulcers, represent approximately 15-30%; and pure arterial insufficiency causes 4-7% of chronic leg ulcers^20^. Most lower limb ulcers are difficult to heal^6^. Their high incidence and morbidity cause severe socioeconomic consequences and burden health services^10^.

### Sociodemographic profile

The age averages shown in the trials are like what is already described in the literature. There is evidence that the occurrence of lower limb ulcers is common among the elderly, usually affecting patients over 65 years of age^21^. Venous leg ulcers that are difficult to heal affect approximately 0.3% to 1% of the general adult population and 3% to 4% of patients aged between 65 and 80 years^21^.

[Fig f1]

**Figure 1 f1:**
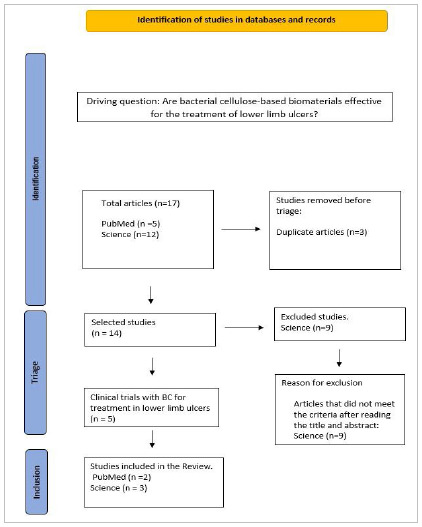
Flowchart of the study inclusion process.

In the populations assessed by Silva et al. (2021), Colenci et al. (2019), and Zanoti et al. (2019), there was a greater predominance of females, corroborating data from other studies that relate the predominance of these changes in females to female physiology and pregnancy, as certain changes predispose to hormonal dysfunction and, consequently, to formation of venous ulcers^22^ ^,^ ^23^. In the study by and Cavalcanti et al. (2017), males constituted most of the sample, 54.5% of the control group and 50% of the experimental one, contrary of what is described in the literature, where the highest occurrence of venous ulcers occur in the female population^22^ ^,^ ^23^.

### Wound characteristics

The main therapeutic effects of the experimental groups were reduction of the wound area, reduction of pain, and decrease in the number of exchanges. The study Zanoti et al. (2019), which used the cellulose membrane cultivated with Gluconacetobacter xylinus bacteria and with the incorporation of Ibuprofen, demonstrated a reduction in the wound area at the end of follow-up from 11.94cm to 11.34cm. The experimental groups that used bacterial cellulose synthesized by Zoogloea sp also showed a reduction in the wound area when compared with the control group. Silva et al. (2021) showed a reduction of 44.18cm^2^ in the wounds’ area, the initial lesions measuring an average of 89.46cm^2^ and, at the end of the follow-up, presented an average of 45.28cm^2^. These results are like a study that used bacterial cellulose dressing for the treatment of pressure injuries, observing a reduction in the mean area of the PI (-14.7cm²) after 30 days of follow-up^15^.

Colenci et al. (2019) found no significant results between the groups regarding decrease in ulcer areas. However, lesions treated with biomembranes showed advantages, such as an improvement in the appearance of the wound bed and reduced exudate, properties that promote faster healing. A clinical trial that compared a biocellulose dressing with a non-adherent VU dressing demonstrated a similar result, without difference in the reduction of the ulcerated area between the treatment groups. However, pain was reduced, and autolytic debridement was faster and more efficient in the biocellulose group than in the standard treatment one^24^.

The presumed objectives of topical VU treatment are to reduce pain and discomfort, eliminate infection and biofilm, help, and promote healing, reduce wound care costs, and improve patients’ quality of life^8^. Pain reduction was reported in all studies. The decrease in pain indicates a lower risk of infection, also allowing the person with the injury to have more freedom, simplifying self-care, and reducing the high operational cost^15^. The post-marketing surveillance study with BC-based epicite hydro dressing obtained through static cultivation of Komagataeibacter xylinus also revealed promising results regarding wound cleansing and pain reduction^25^.

A decrease in the number of dressing changes was also reported, reducing wound handling, and thus avoiding physical injury on the wound bed, a finding also presented in a study using bacterial cellulose as a post-surgical dressing to correct hypospadias^17^. However, reductions in ulcer size and pain were favored by bacterial cellulose.

As observed in the present review, the scarcity of clinical studies that use bacterial cellulose for the treatment of lower limb ulcers makes it difficult to carry out integrative literature review studies that answer questions about the efficiency of this biomaterial.

## CONCLUSION

Bacterial cellulose presents promising results when used as a dressing, favoring the proliferation of granulation tissue, reduction of exudate and, decrease in pain. It should be associated with compression therapy to help venous return in cases of venous insufficiency and can also be used for the treatment of ischemic wounds after revascularization of the lower limbs. The application of BC dressings in humans can be expanded in further studies, benefiting patients, and reducing operational costs associated with the treatment of ulcers.
