# Erratum

**DOI:** 10.1590/0100-6991e-20233536erratatum

**Published:** 2023-06-22

The article “Bacterial cellulose biomaterials for the treatment of lower limb ulcers”, DOI number: 10.1590/0100-6991e-20233536_en, published in the Journal of the Brazilian College of Surgeons 50(1);e20233536.

Where it is:

GLÍCIA MARIA DE OLIVEIRA

- Universidade Federal de Pernambuco, Programa de Pós-graduação em Inovação Terapêutica - Laboratório de Biodispositivos Nanoestruturados, Departamento de Bioquímica - Recife - PE - Brasil

ANTÔNIO OSCAR GOMES FILHO

- Universidade Federal de Pernambuco, Laboratório de Biodispositivos Nanoestruturados, Departamento de Bioquímica - Recife - PE - Brasil

JAIURTE GOMES MARTINS DA SILVA

- Universidade Federal de Alagoas, Campus Arapiraca - Arapiraca - AL - Brasil

ALBERTO GALDINO DA SILVA JUNIOR

- Universidade Federal de Pernambuco, Laboratório de Biodispositivos Nanoestruturados, Departamento de Bioquímica - Recife - PE - Brasil

MARIA DANIELLY LIMA DE OLIVEIRA

- Universidade Federal de Pernambuco, Programa de Pós-graduação em Inovação Terapêutica - Laboratório de Biodispositivos Nanoestruturados, Departamento de Bioquímica - Recife - PE - Brasil

CÉSAR AUGUSTO SOUZA DE ANDRADE

- Universidade Federal de Pernambuco, Programa de Pós-graduação em Inovação Terapêutica - Laboratório de Biodispositivos Nanoestruturados, Departamento de Bioquímica - Recife - PE - Brasil

ESDRAS MARQUES LINS

- Hospital das Clínicas da Universidade Federal de Pernambuco, Departamento de Angiologia e Cirurgia Vascular - Recife - PE - Brasil

It should be:

GLÍCIA MARIA DE OLIVEIRA

- Universidade Federal de Pernambuco, Programa de Pós-graduação em Inovação Terapêutica - Laboratório de Biodispositivos Nanoestruturados, Departamento de Bioquímica - Recife - PE - Brasil

ANTÔNIO OSCAR GOMES FILHO

- Universidade Federal de Pernambuco, Laboratório de Biodispositivos Nanoestruturados, Departamento de Bioquímica - Recife - PE - Brasil

JAIURTE GOMES MARTINS DA SILVA

- Universidade Federal de Alagoas, Campus Arapiraca - Arapiraca - AL - Brasil

ALBERTO GALDINO DA SILVA JUNIOR

- Universidade Federal de Pernambuco, Laboratório de Biodispositivos Nanoestruturados, Departamento de Bioquímica - Recife - PE - Brasil

ESDRAS MARQUES LINS

- Hospital das Clínicas da Universidade Federal de Pernambuco, Departamento de Angiologia e Cirurgia Vascular - Recife - PE - Brasil.

MARIA DANIELLY LIMA DE OLIVEIRA

- Universidade Federal de Pernambuco, Programa de Pós-graduação em Inovação Terapêutica - Laboratório de Biodispositivos Nanoestruturados, Departamento de Bioquímica - Recife - PE - Brasil

CÉSAR AUGUSTO SOUZA DE ANDRADE

- Universidade Federal de Pernambuco, Programa de Pós-graduação em Inovação Terapêutica - Laboratório de Biodispositivos Nanoestruturados, Departamento de Bioquímica - Recife - PE - Brasil

